# *Limosilactobacillus* *reuteri* Urob-7 Alleviates Hyperuricemia by Modulating Uric Acid Metabolism Through Nucleoside Degradation and Xanthine Oxidase Inhibition

**DOI:** 10.3390/foods14213706

**Published:** 2025-10-30

**Authors:** Yizhi Jing, Xiaoyue Bai, Haidong Qian, Yue Wang, Yan Hao, Zhengyuan Zhai, Zhu Zhang, Yanling Hao

**Affiliations:** 1 Key Laboratory of Precision Nutrition and Food Quality, Department of Nutrition and Health, China Agricultural University, Beijing 100193, China; 2 College of Food Science and Nutritional Engineering, China Agricultural University, Beijing 100083, Chinazhaizy@cau.edu.cn (Z.Z.); 3 Teaching and Research Division, School of Chinese Medicine, Hong Kong Baptist University, Kowloon Tong, Kowloon, Hong Kong SAR 999077, China

**Keywords:** *Limosilactobacillus reuteri* Urob-7, hyperuricemia, nucleoside, degradation, xanthine oxidase, gut microbiota

## Abstract

Hyperuricemia (HUA), a metabolic disorder characterized by elevated serum uric acid resulting from imbalanced production and excretion, is associated with gout and other serious health issues. This study aimed to screen out the potential probiotics with HUA-alleviating properties among 20 *Lactobacillus* strains. The results showed that *L. reuteri* Urob-7 exhibited the highest degradation rates for inosine and guanosine (82.10% and 88.78%, respectively) and strong xanthine oxidase (XOD) inhibitory activity (62.86%). In a HUA mouse model induced by inosine, guanosine, and potassium oxonate, *L. reuteri* Urob-7 intervention significantly reduced serum uric acid levels by 46.54%, restoring them to levels similar to control groups, and improved kidney function indicators. Moreover, Urob-7 reduced hepatic XOD activity by 37.6% and downregulated XOD expression in the intestines, decreasing excessive uric acid synthesis. It also significantly inhibited the NF-κB/NLRP3 inflammatory pathway, reducing the expression levels of *NF-κB* and *NLRP3* in the kidneys by 39.3% and 47.6%, respectively. Furthermore, *L. reuteri* Urob-7 increased the abundance of short-chain fatty acid-producing bacteria (e.g., *Ruminococcus* and *Intestinimonas*) while reducing the proportion of pathogenic bacteria (e.g., *Bacteroides* and *Anaerovorax*), thus ameliorating gut microbiota dysbiosis and intestinal barrier dysfunction. In summary, *L. reuteri* Urob-7 effectively relieved HUA by modulating uric acid metabolism, suppressing inflammation, and improving gut microbiota balance. These results highlighted its potential as a promising candidate for HUA.

## 1. Introduction

Hyperuricemia (HUA) is a metabolic disorder characterized by elevated serum uric acid (SUA) concentrations, coupled with imbalanced uric acid synthesis and excretion [1]. HUA is defined as above 7.0 mg/dL (420.0 μmol/L) in males or above 6.0 mg/dL (360.0 μmol/L) in females [2]. HUA is a globally prevalent metabolic disease, with notably high rates in coastal and oceanic regions, such as the United States (21%), Japan (20–25%), and China (15.1%) [3,4,5]. The incidence of HUA is influenced by a variety of factors, including genetics, gender, lifestyle, and diet [6]. Long-term HUA can lead to the deposition of urate crystals in the joints, thereby inducing gout, which is characterized by severe joint pain, inflammation, and swelling [7]. Additionally, HUA is associated with an increased risk of cardiovascular diseases, hypertension, diabetes, and chronic kidney disease [8].

In the human body, urate is the final product of purine metabolism, which involves a multi-step degradation process. Purine nucleosides such as inosine and guanosine are dephosphorylated to form nucleobases, which are then immediately oxidized to produce xanthine, and finally further oxidized to yield urate [9]. Xanthine oxidoreductase (XOD), mainly expressed in the liver and intestines in humans, is a rate-limiting enzyme involved in in vivo uric acid production that catalyzes oxidation from xanthine to uric acid [10,11].The increased activity of XOD can lead to the excessive production of uric acid. In the excretion of uric acid, about 70% of urate is eliminated through the renal system, with the remaining 30% being excreted via the intestinal pathway [12,13]. Urate transporters in intestinal epithelial cells, such as ABCG2, are responsible for transporting uric acid from the blood to the intestinal lumen, where it is directly excreted or decomposed by the intestinal microbiota and then expelled through feces [14].

Current therapeutic approaches for HUA primarily rely on pharmacological interventions, including XOD inhibitors (allopurinol and febuxostat) and uric acid excretion promoters (benzbromarone and rasburicase) [2]. However, these drugs often have significant side effects and poor patient tolerability [15]. In contrast, probiotics offer a safe, cost-effective alternative which can alleviate HUA by reducing uric acid production or increasing uric acid excretion. The metabolites of probiotics, particularly short-chain fatty acids (SCFAs), can reduce lipopolysaccharide (LPS) levels, which in turn decreases inflammatory factors such as IL-1β and alleviates inflammatory responses [16,17]. Specific strains, including *Lactobacillus rhamnosus* CCFM1130, *Lactobacillus rhamnosus* CCFM1131, and *Lactobacillus reuteri* CCFM1132 could promote the production of intestinal SCFAs (especially acetic acid and valeric acid), thereby inhibiting XOD activity. Moreover, probiotic-derived nucleoside hydrolases that can hydrolyze nucleosides, thereby reducing the absorption and metabolism of purines and decreasing uric acid production [18,19]. Additionally, probiotics can restore the balance of the gut microbiota and enhance intestinal barrier disrupted by HUA. Furthermore, probiotics can regulate the expression of uric acid transporters such as URAT1 and ABCG2, thereby modulating the reabsorption and excretion of uric acid [20].

This study aimed to screen out probiotics with nucleoside degradation (inosine and guanosine) and XOD inhibition capabilities in vitro and verify its uric-lowering effects in a high-purine-diet induced HUA mouse model. Specifically, our study delved into the mechanisms of the *L. reuteri* Urob-7 strain, demonstrating its capability to reduce serum uric acid levels through multiple pathways: the degradation of nucleotides, inhibition of xanthine oxidase (XOD) activity, modulation of uric acid transport, and regulation of the gut microbiota. Importantly, we also highlighted its unique potential in ameliorating inflammation. The study provided a foundation for the development of probiotics as a preventive strategy against HUA.

## 2. Materials and Methods

### 2.1. Bacterial Strains and Growth Conditions

In this study, 20 strains of *Lactobacillus* (*Limosilactobacillus fermentum*, *Lactiplantibacillus plantarum*, *Limosilactobacillus reuteri*, *Lacticaseibacillus rhamnosus*) isolated from different traditional fermented food stored at −80 °C were selected as candidate strains (Table 1). The strains were inoculated into 2% (*v*/*v*) de Man, Rogosa, and Sharp (MRS) medium (Beijing Aoboxing Biotech Co., Ltd., Beijing, China) and subcultured three times at 37 °C for 24 h.

### 2.2. Determination of Nucleoside Degradation Ability In Vitro

The nucleoside degradation ability of the strains in vitro was determined according to the method described by Xiao Y et al. [21]. After three generations of cultivation, the culture solution was centrifuged at 8000 rpm, 4 °C for 5 min to collect the bacterial pellet. Then, the sediments were washed by sterile phosphate-buffered saline (PBS, pH = 7.2) for three times and re-suspended in 1 mL of a 0.5 g/L inosine-guanosine standard solution and incubated at 37 °C for 60 min. Then, the solution was centrifuged and added with 0.1 mol/L HClO_4_ (9:1, *v*/*v*) to terminate further degradation. A 10 µL of the mixture was filtered through a 0.22-μm filters and injected into the high-performance liquid chromatography (HPLC) system (LC-20A, Shimadzu Corporation, Kyoto, Japan) to analyze the remaining contents of inosine and guanosine. PBS was used as the negative control to minimize background interference and to assess the experimental system’s stability.

HPLC conditions: chromatographic column, INERTSUSTAIN AX-C18, 5 μM, 4.6 × 250 mm; mobile phase, an isocratic gradient of 10 mM KH_2_PO_4_ solution to methanol (95:5, *v*/*v*); flow rate, 1 mL/min; column temperature, 30 °C; UV detection wave length, 254 nm; total retention time, 20 min. The external standard method was employed for the quantitative determination of inosine and guanosine contents using an HPLC system.

### 2.3. Determination of In Vitro XOD Inhibitory Activity

A 300 μL of the bacterial fermentation supernatant was combined with equal volume of XOD solution (0.05 U/mL) or 1 × PBS and incubated at 25 °C for 30 min. Then, a 300 μL of the above sample solution was added with 450 μL xanthine (0.48 mM) solution and incubated at 25 °C for 1 h. After centrifugation (8000 rpm, 25 °C, 10 min), the optical density (OD) value at 290 nm was determined using a Microplate reader. XOD inhibitory activity is calculated according to the following formula [22].

Inhibition rate (%) = [1 − (A1 − A2)/(A3 − A4)] * 100%

where A1 represents the absorbance of the test samples with enzyme; A2 represents the absorbance of the test solution without enzyme; A3 represents the absorbance of the blank group with 1 × PBS with enzyme; A4 represents the absorbance of 1 × PBS without enzyme added.

### 2.4. Animal Experiments

Three-week-old SPF Kunming male mice (Beijing Vital River Laboratory Animal Technology Co., Ltd., Beijing, China) weighing 20 ± 2 g were obtained and acclimated for seven days under conditions including a room temperature of 21 ± 2 °C, humidity maintained at 50 ± 10%, and a 12 h light/dark cycle. They were randomly divided into three groups (*n* = 6): the control group (CK), the HUA model group (MOD), and the Urob-7 intervention group (Urob-7). All groups were fed a basic maintenance diet for mice (Sipeifu Biotechnology Co., Ltd., Beijing, China) and provided with purified drinking water. The HUA model was established by intragastric administration of 250 mg/kg potassium oxonate (OP), 400 mg/kg inosine (INO), and 400 mg/kg guanosine (GUO), administered as a dispersion in 0.5% (*w*/*v*) carboxymethylcellulose sodium (CMC-Na). To assess its preventive potential, *Limosilactobacillus reuteri* Urob-7 was administered intragastrically to the Urob-7 group at a dose of 10^9^ CFU/d starting from the modeling initiation. The CK group received an equal volume of 1 × PBS and 0.5% (*w*/*v*) CMC-Na daily. On the 7th, 14th, and 21st days, blood samples were collected from the mice’s orbital sinus, and serum uric acid levels were quantified using a uric acid detection kit (Nanjing Jiancheng, Nanjing, China). On the 21st day following the blood sampling, the mice were anesthetized with isoflurane and euthanized by cervical dislocation. Feces, liver, kidney, spleen, and intestinal tissues were collected and stored at −80 °C until analysis. All experiments were approved by the Animal Care and Use Committee of China Agricultural University and conformed to the Guide for the Care and Use of Laboratory Animals (Approval number: AW42604202-5-1).

### 2.5. Histopathological Analysis

The intestinal tissues (ileum and colon), liver and kidney tissues samples were fixed in 4% paraformaldehyde for 24 h, then embedded in paraffin. Sections of 5 μm were cut, dewaxed, rehydrated and hematoxylin and eosin (H&E)-stained. Histopathology and inflammation were assessed via optical microscopy. Villus height and crypt depth were measured using ImageJ 1.51 (NIH, Bethesda, MD, USA).

### 2.6. Liver XOD Activity Measurement

The liver tissue was homogenized at low temperature and centrifuged to obtain the supernatant, and UA synthesis in the liver was evaluated by the activity of xanthine oxidase (Solarbio, BC 1095, Beijing, China) according to the manufacturer’s instructions.

### 2.7. Serum Biochemical Indicators Examination

Blood samples were left for 2 h after collection and centrifuged (3500 rpm, 4 °C, 15 min) to obtain serum samples. Serum uric acid (UA), creatinine (Cr) and urea nitrogen (BUN) were detected with a commercial assay kit (Jiancheng, Nanjing, China).

### 2.8. Immunofluorescence Staining

Sections were deparaffinized in xylene, rehydrated with graded ethanol, and underwent antigen retrieval in citrate buffer (pH 6.0) via microwave heating for 20 min. After permeabilization with 1% Triton X-100 and blocking with 5% BSA, sections were incubated with primary antibodies, followed by Alexa Fluor 488-labeled secondary antibodies (1:1000). Nuclei were stained with DAPI, and sections were sealed with an antifluorescence quenching agent. Fluorescence microscopy was used for observation, and ImageJ software quantified ABCG2 expression and localization in the tissue sections.

### 2.9. Quantitative Reverse Transcription PCR (qRT-PCR)

The tissue samples (50 mg) were homogenized and mixed with 1 mL AG RNAex Pro reagent (Accurate Biotechnology Hunan Co., Ltd., Changsha, China) for total RNA extraction. The RNA was dissolved in RNase-free water and stored at −80 °C. Then, 1 µg of total RNA was reverse-transcribed into cDNA using the Evo M-MLV RT Mix Kit with gDNA Clean. RT-qPCR was performed with the SYBR Green Premix Pro Taq Hs qPCR kit on a QuantStudio™5 system (Thermo Fisher, Wilmington, MA, USA). Primers are listed in Table 2. Target gene expression was quantified relative to β-actin using the 2^−∆∆CT^ method.

### 2.10. Gut Microbiota 16S rRNA Gene Analysis

Microbial genomic DNA was extracted from fecal samples using the FastPure Stool DNA Isolation Kit (MJYH, Shanghai, China). DNA quality and concentration were assessed via 1.0% agarose gel electrophoresis and a NanoDrop^®^ ND-2000 spectrophotometer. The V3-V4 region of the 16 S rRNA gene was amplified using primers 338F and 806R with PCR conditions: 95 °C for 3 min, 27 cycles of 95 °C for 30 s, 55 °C for 30 s, 72 °C for 45 s, and a final extension at 72 °C for 10 min. PCR products were purified, quantified, and pooled equimolarly for paired-end sequencing on the Illumina NextSeq 2000 platform by Majorbio Bio-Pharm Technology Co. Ltd. (Shanghai, China) Bioinformatics analysis was performed using the Majorbio Cloud platform, including rarefaction curves, alpha diversity indices, PCoA analysis, and identification of differentially abundant taxa.

### 2.11. Statistical Analysis

GraphPad Prism 9.5 was used for statistical analysis. The results were expressed as mean ± SEM. One-way ANOVA with Tukey’s multiple comparisons test was used to identify the statistically significant differences. A *p*-value < 0.05 was considered to indicate statistical significance.

## 3. Results

### 3.1. Screening of Lactobacillus Strains with Uric Acid-Lowering Activity In Vitro

**Figure 1:**
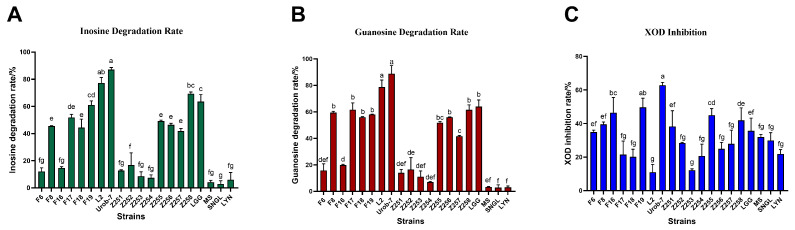
Nucleoside degradation and XOD inhibition ability of 20 *Lactobacillus* strains in vitro. (**A**) Degradation rate of inosine. (**B**) Degradation rate of guanosine. (**C**) Inhibition rate of XOD activity. *n* = 3. Data were presented as mean ± SEM. In each subfigure, different letters above the bars indicate significant differences between groups (*p* < 0.05). Bars with the same letter are not significantly different.

The degradation abilities of inosine and guanosine by 20 *Lactobacillus* strains were analyzed using HPLC. The results showed that *L. reuteri* Urob-7 exhibited the highest inosine and guanosine degradation rates with 82.10% and 88.78%, followed by *L. reuteri* L2 with rates of 75.23% and 78.67% (Figure 1A,B). However, *L. reuteri* L2 showed relatively low inhibitory activity against XOD at only 10.96%, while Urob-7 the highest inhibition rate among the tested strains at 62.86% (Figure 1C). Therefore, *L. reuteri* Urob-7 was selected to explore its uric acid-lowering ability in vivo.

### 3.2. L. reuteri Urob-7 Alleviated Hyperuricemia by Decreasing Serum UA Levels

**Figure 2:**
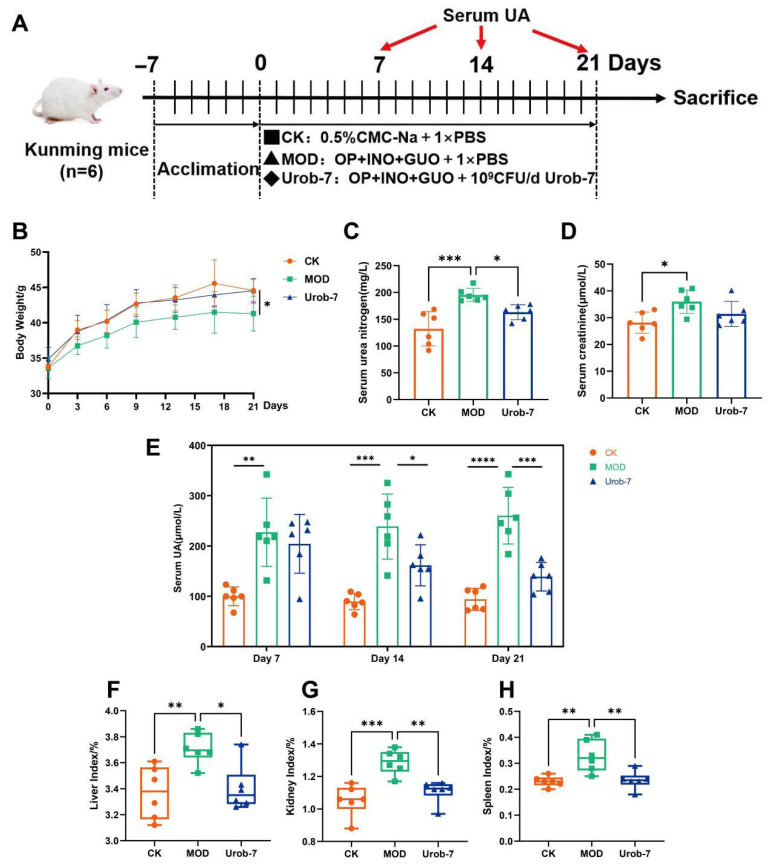
Effects of *L. reuteri* Urob-7 on symptoms of HUA. (**A**) Schematic diagram of the animal experiment. (**B**) Changes in body weight. (**C**) Serum BUN levels. (**D**) Serum Cr levels. (**E**) Serum uric acid levels during a 21-day period. (**F**–**H**) Organ coefficients of livers, kidneys and spleens. *n* = 6 mice per group. Data were presented as mean ± SEM. *p* values were determined by one-way ANOVA followed by Tukey’s test. * *p* < 0.05, ** *p* < 0.01, *** *p* < 0.001, and **** *p* < 0.0001.

To investigate the effects of Urob-7 on alleviating HUA, a mouse model combined with nucleosides and potassium oxonate was established (Figure 2A). After 21 days’ administration, the body weight of the MOD group decreased by 7.19% compared to the CK group, while there was no significant difference between the Urob-7 group and CK group (Figure 2B). Serum BUN and CR levels were significantly elevated in the MOD group compared to the CK group. Urob-7 intervention restored the BUN levels to that of the CK group, though CR levels remained unchanged relative to the MOD group (Figure 2C,D). Notably, the serum uric acid level in the MOD group remained approximately 2-3-fold higher than that in the CK group. In contrast, the serum uric acid level in the Urob-7 group showed a downward trend from day 7 to day 14 and decreased to nearly the similar level as the CK group at day 21. Compared with CK group, the liver, kidney, and spleen indexes of the MOD group were significantly higher than those of the CK group, increasing by 10.15%, 22.32%, and 42.74%, respectively (Figure 2F–H). The intervention with *L. reuteri* Urob-7 effectively reduced the organ coefficients to levels comparable to those of the CK group. These results indicated that *L. reuteri* Urob-7 had therapeutic potential for HUA by reducing serum uric acid, mitigating organ swelling, and modulating kidney function.

### 3.3. L. reuteri Urob-7 Ameliorated the Liver, Kidney, and Intestinal Structural Damage

**Figure 3:**
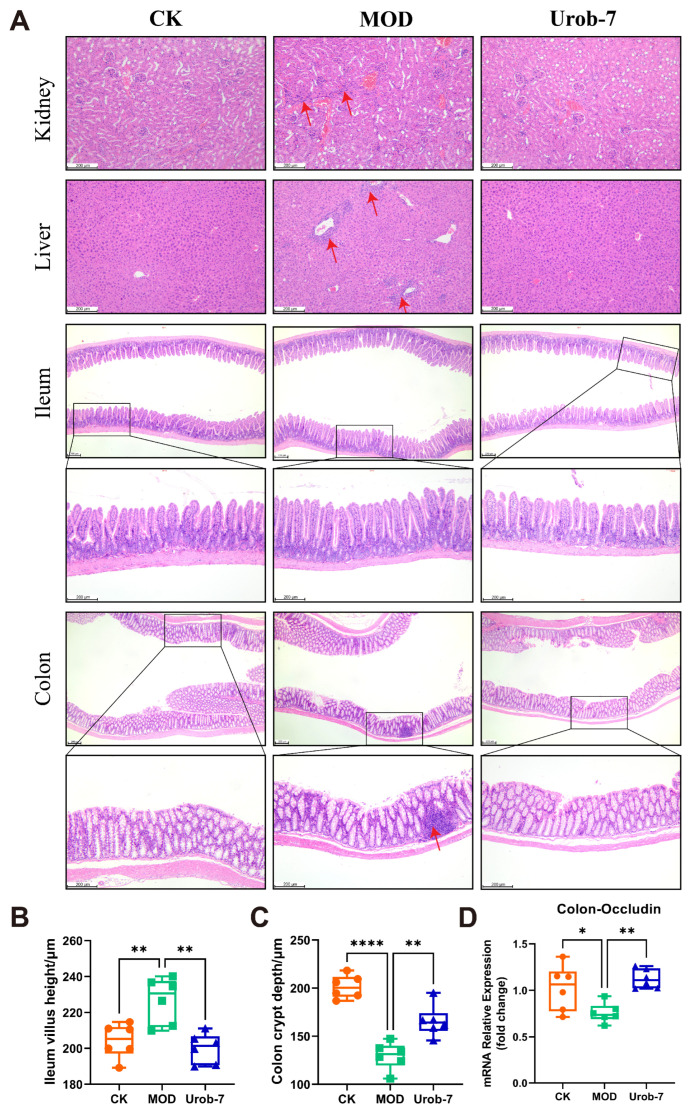
Effects of *L. reuteri* Urob-7 on histopathological damage to the kidney, liver, and intestines in HUA mice. (**A**) Representative images of H&E staining in the kidney, liver, ileum and colon. The red arrows represent inflammatory cell infiltration. (**B**) The villi height of ileum. (**C**) The crypt depth of colon. (**D**) Expression levels of Occludin mRNA in the colon. *n* = 6 mice per group. Data were presented as mean ± SEM. *p* values were determined by one-way ANOVA followed by Tukey’s test. * *p* < 0.05, ** *p* < 0.01, and **** *p* < 0.0001.

Histopathological analysis (Figure 3A) showed that MOD group mice exhibited marked renal damage, characterized by incomplete glomerular morphology, capillary loop expansion, Bowman’s capsule swelling, and mild inflammatory infiltration. Hepatic sections from MOD mice showed turbid cytoplasm, red blood cell stasis in sinusoids, significant inflammatory infiltration, and variable-sized fat vacuoles. As for the intestines, the colon structure in MOD group mice was distorted with shortened crypts, and marked inflammatory infiltration. Compared with the CK group, the MOD group exhibited a significant increase in ileum villus height. In contrast, Urob-7 group mice showed less severe kidney damage, with neatly arranged proximal tubular epithelial cells and clearer cytoplasm. Urob-7 intervention also reduced hepatic fat deposition and inflammation, leading to more regular and uniform hepatocyte morphology. Compared with the MOD group, ImageJ analysis further confirmed that mice in the Urob-7 group exhibited significantly reduced ileum villus height and increased colon crypt depth (Figure 3B,C). Additionally, the expression level of Occludin was higher in the Urob-7 group than in the MOD group (Figure 3D), indicating that Urob-7 could ameliorate intestinal pathological changes induced by HUA. Overall, these findings suggested that *L. reuteri* Urob-7 intervention effectively alleviated HUA-induced pathological changes and inflammation in the liver, kidneys, and intestines.

### 3.4. L. reuteri Urob-7 Regulated Uric Acid Re-Absorption and Excretion Transporter

**Figure 4:**
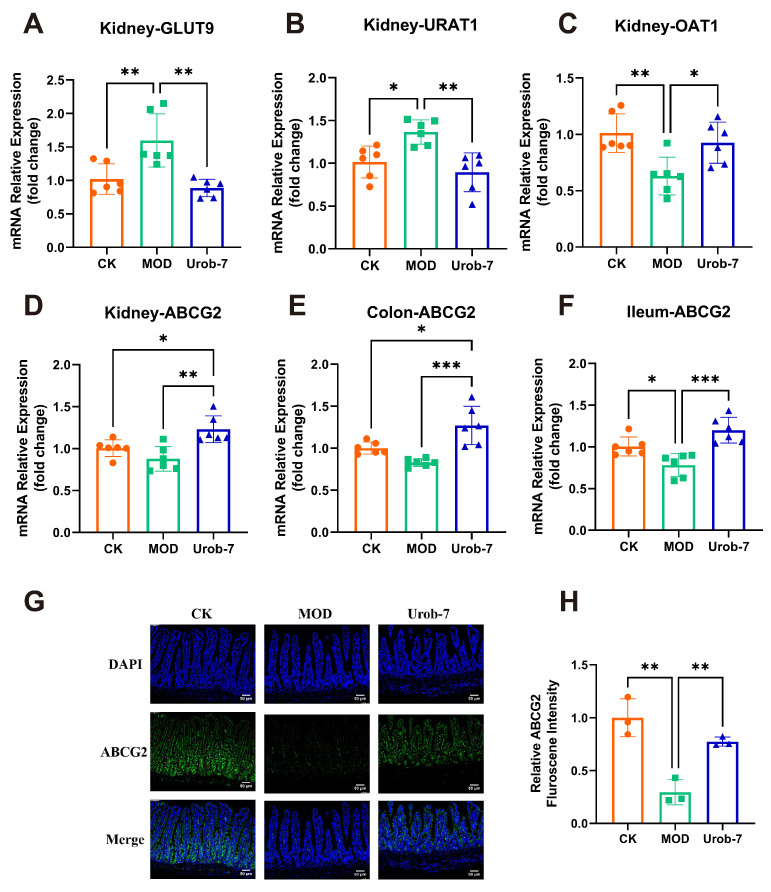
Effects of *L. reuteri* Urob-7 on uric acid transporter. The mRNA expression levels of (**A**) *GLUT9*, (**B**) *URAT1*, (**C**) *OAT1* and (**D**) *ABCG2* in the kidney. The expression levels of *ABCG2* in the colon (**E**) and ileum (**F**). *n* = 6 mice per group. (**G**) Representative immunofluorescence staining images of ABCG2 (green). Nuclei were stained by DAPI (blue). Scale bar means 50 μm. (**H**) Quantification of ABCG2 fluorescence intensity. *n* = 3 mice per group. Data were presented as mean ± SEM. *p* values were determined by one-way ANOVA followed by Tukey’s test. * *p* < 0.05, ** *p* < 0.01, and *** *p* < 0.001.

To investigate the effects of *L. reuteri* Urob-7 on uric acid metabolism, re-absorption transporters (*GLUT9* and *URAT1*) and excretion transporters (*ABCG2* and *OAT1*) were analyzed. The results showed that mRNA levels of *GLUT9* and *URAT1* significantly increased by 56.57% and 34.45%, while mRNA levels of OAT1 and ABCG2 decreased by 37.62% and 12.61% in the MOD group (Figure 4A–D). Moreover, *ABCG2* expression in the ileum and colon of MOD group also significantly decreased (Figure 4E,F). *L. reuteri* Urob-7 intervention reversed these changes by significantly down-regulating the mRNA expression of *GLUT9* and *URAT1* and upregulating *OAT1* and *ABCG2* in the kidney. Furthermore, *ABCG2* expression in the intestinal tissues was significantly upregulated. Immunofluorescence analysis confirmed that ABCG2 protein expression in the ileum of the Urob-7 group was significantly higher than in the MOD group (Figure 4G,H). These findings suggested that *L. reuteri* Urob-7 effectively promoted uric acid excretion and reduced uric acid reabsorption by regulating the expression of these key transporters, thereby alleviating HUA.

### 3.5. L. reuteri Urob-7 Ameliorated NLRP3 Inflammatory Response and Inhibited Uric Acid Synthesis

**Figure 5:**
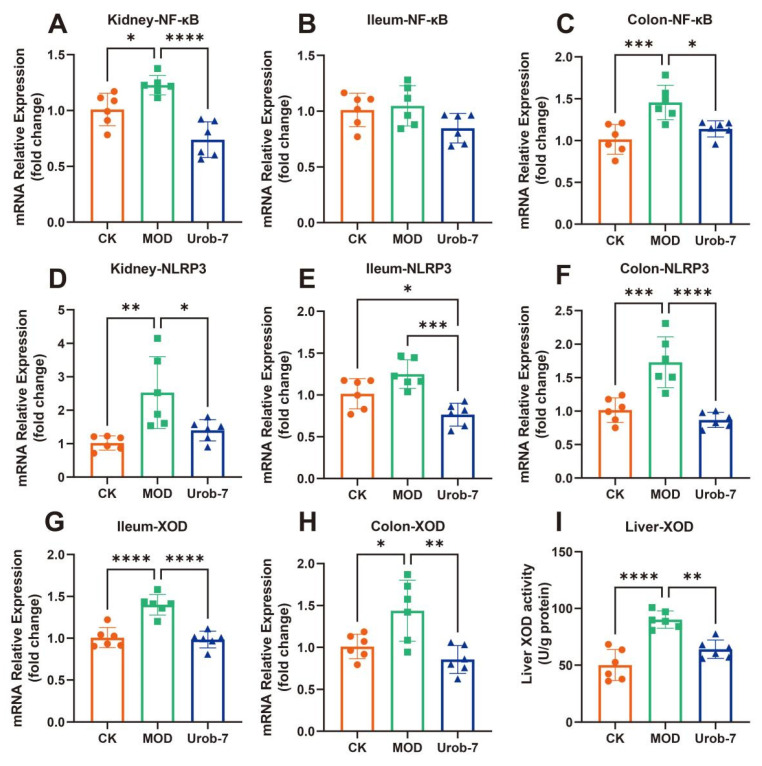
Effects of *L. reuteri* Urob-7 on inflammatory response and XOD in vivo. The mRNA expression levels of (**A**) Kidney *NF-κB*, (**B**) Ileum *NF-κB*, (**C**) Colon *NF-κB*, (**D**) Kidney *NLRP3*, (**E**) Ileum *NLRP3*, (**F**) Colon *NLRP3*, (**G**) Ileum XOD, (**H**) Colon XOD. (**I**) Liver XOD activity. *n* = 6 mice per group. Data were presented as mean ± SEM. *p* values were determined by one-way ANOVA followed by Tukey’s test. * *p* < 0.05, ** *p* < 0.01, *** *p* < 0.001, and **** *p* < 0.0001.

The expressions of inflammatory signaling pathways and key enzymes in uric acid metabolism were evaluated in three groups of mice (Figure 5A–F). The results showed that the gene expression of *NF-κB* was significantly upregulated in the kidney and colon of the MOD group, increasing by 39.30% and 43.63% respectively compared with the CK group. Similarly, the mRNA expression levels of *NLRP3* in the kidney and colon of MOD group mice were also significantly upregulated. However, there were no significant differences in *NF-κB* expression among the three groups in the ileum, and significant differences in *NLRP3* expression were only observed between the Urob-7 group and the other two groups. Notably, *L.reuteri* Urob-7 intervention effectively down-regulated the gene expression at the mRNA level, restoring the levels of *NF-κB* and *NLRP3* in the kidney and colon to levels similar to those of the CK group.

XOD gene expression was significantly upregulated in both the colon and ileum in MOD group, but Urob-7 intervention reversed this effect (Figure 5G,H). Meanwhile, XOD activity in the liver was significantly increased in the MOD group, while oral administration of *L.reuteri* Urob-7 markedly reduced XOD activity in the livers of HUA mice (Figure 5I). These findings suggested that Urob-7 alleviated HUA by suppressing NLRP3-mediated inflammation and modulating XOD expression and activity, thereby reducing uric acid production and its associated inflammatory responses.

### 3.6. L. reuteri Urob-7 Reshaped Gut Microbiota Composition in Mice with HUA

**Figure 6:**
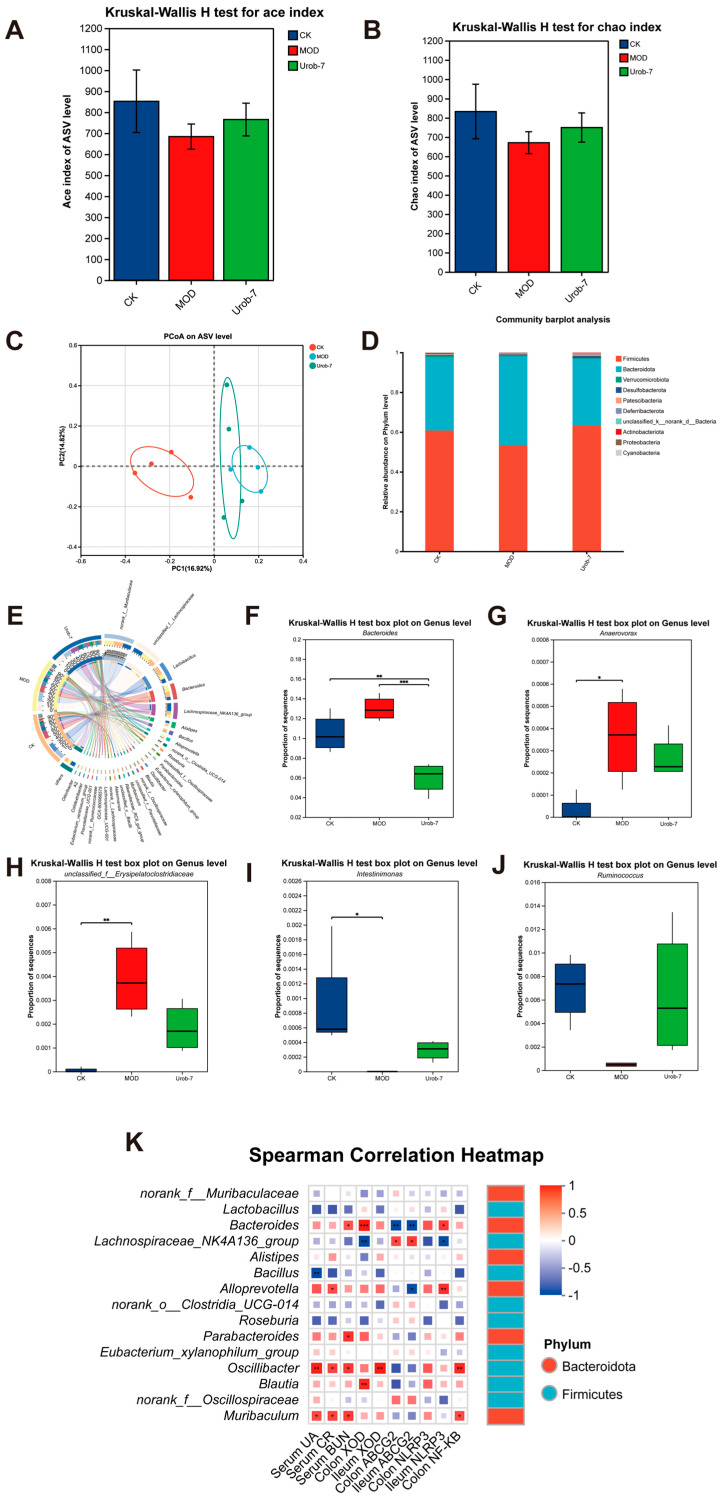
Effects of *L. reuteri* Urob-7 on gut microbiota composition in HUA mice. (**A**) Ace index of Alpha diversity. (**B**) Chao index of Alpha diversity. (**C**) Principal coordinates analysis (PCoA) plot on ASV level. (**D**) Relative abundance of microbiota at the phylum level. (**E**) Circos diagram for community diversity analysis at the genus level. (**F**–**J**) The relative abundance of *Bacteroides*, *Ruminococcus*, *Intestinimonas*, *unclassified_f__Erysipelatoclostridiaceae* and *Anaerovorax*. (**K**) Spearman correlation analysis between microbiota at the genus level and biochemical parameters. Box colors indicated the level of correlation coefficients. *n* = 6 mice per group. Data were presented as mean ± SEM. *p* values were determined by one-way ANOVA followed by Tukey’s test. * *p* < 0.05, ** *p* < 0.01, and *** *p* < 0.001.

To illustrate the effect of *L. reuteri* Urob-7 on the gut microbiota composition in HUA mice, the diversity and abundance of gut microbiota were detected in fecal samples via 16S rRNA sequencing. Compared with the CK group, the Ace index and Chao index of mice in the MOD group decreased but did not exhibit a significant difference. The Ace index and Chao index of the Urob-7 group increased compared with those of the MOD group mice, but did not restore to the same level as the CK group (Figure 6A,B). A principal coordinates analysis (PCoA) showed a significant difference between the CK and MOD groups at the ASV level (PCoA 1 = 16.92%, PCoA 2 = 14.82%) (Figure 6C). Moreover, the Urob-7 group exhibited a trend towards the separation and clustering of microbiota structures from the MOD group, indicating *L. reuteri* Urob-7 treatment may change the gut microbiota composition. In addition, compared to the CK group, the relative abundance of *Firmicutes* and *Actinobacteriota* was significantly reduced, while that of *Bacteroidetes* was significantly increased in the MOD group (Figure 6D). The administration of *L. reuteri* Urob-7 reversed this imbalance and restored the ratio of *Bacteroidetes* to *Firmicutes* (Bac/Firm Ratio) to the level of the CK group. At the genus level, the circus plot analysis showed that *norank_f__Muribaculaceae*, *unclassified_f__Lachnospiraceae*, *Lactobacillus*, *Bacteroides*, *Lachnospiraceae_NK4A136_group*, and *Alistipes* are the shared core genera in the three groups of mice (Figure 6E). Furthermore, compared with the CK group, the relative abundance of *Bacteroides*, *unclassified_f__Erysipelatoclostridiaceae*, and *Anaerovorax* significantly increased (*p* < 0.05) in the MOD group, while the relative abundance of *Ruminococcus* and *Intestinimonas* significantly decreased (*p* < 0.05) (Figure 6F–J). The Spearman correlation heatmap analysis showed that the relative abundance of *Bacteroides* was positively correlated with serum biomarker concentrations (UA, CR, and BUN), XOD expression, and inflammation-related genes, while negatively correlated with ABCG2 expression in the ileum and colon (Figure 6K). These results suggested that Urob-7 intervention effectively reversed the HUA-induced imbalance in gut microbiota.

## 4. Discussion

Purine bases in food are mainly present in the form of nucleoproteins. During digestion, gastric acid initiates the breakdown of nucleoproteins into nucleic acids and proteins. In the small intestine, pancreatic nucleases further degrade nucleic acids into mononucleotides. These mononucleotides are subsequently hydrolyzed by enzymes such as 5’-nucleotidase to produce nucleosides and phosphate [23]. The purine-containing nucleosides are absorbed by intestinal epithelial cells and are eventually metabolized into uric acid in the liver [24]. Thus, reducing the absorption of nucleosides into the bloodstream is crucial for controlling blood uric acid levels. As precursors of uric acid synthesis, inosine and guanosine can be hydrolyzed into purine bases by enterocytes. Probiotics can decrease the host’s nucleoside absorption, thereby lowering uric acid production. Li et al. [25] discovered three genes annotated as ribonucleoside hydrolase (*RihA*, *RihB* and *RihC*) involved in the conversion of inosine and guanosine in the *L. plantarum* genome. Similarly, *L. reuteri* has been reported to harbor the ribonucleoside hydrolase gene *RihC* [26]. The protein encoded by this gene can efficiently degrade inosine, reducing its intestinal circulation and hepatic accumulation. XOD is the rate-limiting enzyme in uric acid synthesis, and inhibiting its activity can also significantly reduce uric acid production. In this study, *L. reuteri* Urob-7 demonstrated high nucleoside degradation and XOD inhibiting activities. As a probiotic naturally present in the human intestinal tract, *L. reuteri* exhibits tolerance to gastrointestinal fluids [27]. Notably, we report for the first time that *L. reuteri* Urob-7 demonstrates robust nucleoside hydrolase activity as well as potent XOD inhibition.

HUA is often accompanied by inflammatory responses [28]. Monosodium urate (MSU) crystals can activate the NF-κB pathway via TLR2/4-MyD88 signaling, inducing NLRP3 and pro-IL-1β expression. Meanwhile, MSU-induced lysosomal disruption or ROS production triggers NLRP3 inflammasome assembly, activating caspase-1 to cleave pro-IL-1β into mature IL-1β. The released IL-1β binds to IL-1 receptors (IL-1R), reactivating the MyD88/NF-κB pathway and forming a positive feedback loop [29,30]. Increased XOD activity leads to excessive uric acid production and MSU crystal formation, further aggravating the inflammatory response [31]. Recently, probiotics have shown potential in alleviating HUA by modulating NLRP3 inflammasome activation and inflammatory factor release. *Lactobacillus acidophilus* F02 suppressed urate-induced upregulation of pro-IL-1β, IL-1β, NLRP3, ASC, and caspase-1 expression, thereby mitigating HUA-induced hepatorenal injury [32]. In this study, *L. reuteri* Urob-7 effectively suppresses the NLRP3 inflammasome pathway, thereby mitigating inflammation associated with hyperuricemia. This integrated approach offers a more comprehensive strategy for hyperuricemia management compared to probiotics that act through single mechanistic actions. For example, *L. rhamnosus* Fmb14 has been shown to alleviate hyperuricemia-induced hepatocyte pyroptosis by inhibiting the NLRP3 inflammasome cascade [33], but it does not exhibit notable nucleoside degradation or XOD inhibitory capabilities. Similarly, *L. plantarum* has been reported to reduce uric acid levels by enhancing colonic uric acid excretion, yet its effect on XOD activity and nucleoside metabolism remains limited [20]. The multifaceted mechanism of *L. reuteri* Urob-7 makes it a promising candidate for the development of natural therapeutics against hyperuricemia, offering a broader spectrum of benefits including uric acid metabolism regulation, inflammation suppression, and gut microbiota restoration.

HUA is closely linked to gut microbiota dysbiosis [34]. Compared to healthy individuals, the proportion of *Firmicutes* decreases, while *Bacteroidota* and *Proteobacteria* increase at the phylum level in HUA patients [35]. At the genus level, *Blautia*, *Collinsella*, and *Turicibacter* are positively correlated with HUA, while SCFAs-producing bacteria like *Alistipes*, *Roseburia*, *Parabacteroides*, and *Clostridium* are negatively correlated [36]. These SCFA-producing bacteria play critical roles in maintaining gut barrier integrity and modulating inflammatory pathways, and their reduced abundance may disrupt intestinal homeostasis, exacerbate metabolic disorders, and promote systemic inflammation [37,38]. In this study, Urob-7 intervention increased the relative abundance of *Firmicutes* and *Actinobacteriota* and decreased *Bacteroidota* at the phylum level. Notably, *Actinobacteriota* may alleviate HUA-associated inflammation through Treg cell activation or NF-κB pathway suppression [39]. At the genus level, the relative abundance of *Bacteroides*, *unclassified_f__Erysipelatoclostridiaceae*, and *Anaerovorax* decreased significantly in the Urob-7 group, while the relative abundance of *Ruminococcus* and *Intestinimonas* increased. *Bacteroides* has been implicated in the metabolic conversion of purines to uric acid. A reduction in the abundance of *Bacteroides* may suppress this metabolic pathway, thereby potentially mitigating uric acid accumulation in the host [34]. Its reduced abundance may mitigate urate accumulation and enhance gut barrier integrity, thereby curbing bacterial translocation and inflammatory cytokine release [40]. Previous studies have shown that the bacterial genera Ruminococcus and Intestinimonas are capable of degrading resistant starch into butyrate via specific metabolic pathways [41]. Butyrate, by activating the PPARγ pathway, exerts anti-inflammatory effects, upregulates gut ABCG2 transporter expression, and facilitates uric acid excretion [42,43]. Collectively, these functional shifts in the gut microbiota highlight potential mechanisms through which microbiota influence uric acid metabolism and systemic inflammation.

Studies have demonstrated a significant positive correlation between serum uric acid concentrations and pro-inflammatory cytokines, including *IL-6* and *IL-1β*, in elderly inpatients [44]. This aligns with the growing body of evidence linking uric acid metabolism to inflammatory responses, which is of particular relevance in the context of hyperuricemia and associated co-morbidities. By modulating uric acid levels and mitigating inflammation, *L. reuteri* Urob-7 may offer a synergistic approach to managing hyperuricemia, with potential implications for improving patient outcomes in clinical settings. This integrated mechanism highlights the probiotic’s promise in addressing the complex interplay between metabolic dysfunction and inflammation, as often observed in conditions like hyperuricemia. Furthermore, it underscores the need for broader exploration of such multi-targeted strategies in the development of novel therapeutic interventions. In conclusion, *L. reuteri* Urob-7 effectively alleviated HUA by degrading nucleosides, inhibiting XOD activity, reducing inflammation, and restoring gut microbiota homeostasis.

## Figures and Tables

**Table 1:** Strains used in this work.

Strain Name	Latin Name	Strain Source
F6	*Limosilactobacillus fermentum*	Cow milk
F8	*Limosilactobacillus fermentum*	Buffalo milk
F16	*Limosilactobacillus fermentum*	Traditional Yunnan fermented pork
F17	*Limosilactobacillus fermentum*	Traditional Yunnan fermented pork
F18	*Limosilactobacillus fermentum*	Suan-shui (acidic whey from Rubing cheese)
F19	*Limosilactobacillus fermentum*	Suan-shui (acidic whey from Rubing cheese)
L2	*Limosilactobacillus reuteri*	Suan-shui (acidic whey from Rubing cheese)
Urob-7	*Limosilactobacillus reuteri*	Suan-shui (acidic whey from Rubing cheese)
Z251	*Lactiplantibacillus plantarum*	Cow milk
Z252	*Lactiplantibacillus plantarum*	Cow milk
Z253	*Lactiplantibacillus plantarum*	Cow milk
Z254	*Lactiplantibacillus plantarum*	Cow milk
Z255	*Lactiplantibacillus plantarum*	Pickled vegetables
Z256	*Lactiplantibacillus plantarum*	Pickled vegetables
Z257	*Lactiplantibacillus plantarum*	Pickled vegetables
LGG	*Lacticaseibacillus rhamnosus*	From Wageningen University
MS	*Lacticaseibacillus rhamnosus*	Air-dried sausage
SNGL	*Lacticaseibacillus rhamnosus*	Air-dried sausage
LYN	*Lacticaseibacillus rhamnosus*	Air-dried sausage

**Table 2:** qRT-PCR primers used in this work.

Gene	Forward Primer (5’-3’)	Reverse Primer (5’-3’)
*β-actin*	GTGACGTTGACATCCGTAAAGA	GCCGGACTCATCGTACTCC
*Glut9*	TTGCTTTAGCTTCCCTGATGTG	GAGAGGTTGTACCCGTAGAGG
*Oat1*	CTGATGGCTTCCCACAACAC	GTCCTTGCTTGTCCAGGGG
*Urat1*	TCACCACCCAGAACATGCTG	GGAGACGGCCAGAAGAACAT
*Abcg2*	GAACTCCAGAGCCGTTAGGAC	CAGAATAGCATTAAGGCCAGGTT
*Xod*	ATGACGAGGACAACGGTAGAT	TCATACTTGGAGATCATCACGGT
*Nlrp3*	ATTACCCGCCCGAGAAAGG	TCGCAGCAAAGATCCACACAG
*Nf-κb*	ATGGCAGACGATGATCCCTAC	TGTTGACAGTGGTATTTCTGGTG
*Il-1β*	GCAACTGTTCCTGAACTCAACT	ATCTTTTGGGGTCCGTCAACT

## Data Availability

The original contributions presented in this study are included in the article. Further inquiries can be directed to the corresponding author.
